# COVID-19 experiences predicting high anxiety and depression among a sample of BRCA1/BRCA2-positive women in the US

**DOI:** 10.1038/s41598-021-04353-x

**Published:** 2021-12-30

**Authors:** Kate E. Dibble, Avonne E. Connor

**Affiliations:** 1 Department of Epidemiology, Johns Hopkins Bloomberg School of Public Health, 615 N. Wolfe Street, Baltimore, MD 21205 USA; 2 Department of Oncology, Johns Hopkins Sidney Kimmel Comprehensive Cancer Center, Baltimore, MD 21205 USA

**Keywords:** Breast cancer, Genetics, Psychology

## Abstract

During the COVID-19 pandemic, breast and ovarian cancer survivors experienced more anxiety and depression than before the pandemic. Studies have not investigated the similarities of this trend among *BRCA1/2*-positive women who are considered high risk for these cancers. The current study examines the impact of COVID-19 experiences on anxiety and depression in a sample of *BRCA1/2*-positive women in the U.S. 211 *BRCA1/2*-positive women from medically underserved backgrounds completed an online survey. Adjusted odds ratios (aORs) and 95% confidence intervals (CIs) were estimated using multivariable logistic regression for associations between COVID-19 experiences and self-reported anxiety and depression stratified by demographic factors. Overall, women who reported COVID-19 stigma or discrimination (aOR, 5.14, 95% CI [1.55, 17.0]) experienced significantly more depressive symptoms than women who did not report this experience. Racial/ethnic minority women caring for someone at home during COVID-19 were 3.70 times more likely (95% CI [1.01, 13.5]) to report high anxiety while non-Hispanic white women were less likely (aOR, 0.34, 95% CI [0.09, 1.30], *p* interaction = 0.011). To date, this is the first study to analyze anxiety and depression considering several COVID-19 predictors among *BRCA1/2*-positive women. Our findings can be used to inform future research and advise COVID-19-related mental health resources specific to these women.

## Introduction

One in eight women will be diagnosed with breast cancer in their lifetime, but only 5–10% of these women have a *BRCA1* and/or *BRCA2* (BReast CAncer) genetic mutation^1^. Although rare, these mutations occur on dominant genes, indicative of a 50% inheritance rate, and therefore, occur within biological family units and often co-occur with other rarer cancer-specific mutations such as *ATM*, *CDH1*, *CHEK2*, *PALB2*, *PTEN*, *STK11*, and *TP53*^2^. Due to these mutations, women have an increased risk of breast and ovarian cancers^3^, living with a cumulative breast cancer risk of 72% among *BRCA1* and 69% among *BRCA2* carriers^4^. Ovarian cancer risk is also elevated by the presence of these mutations, with one study finding that ovarian cancer occurs in an estimated 44% of *BRCA1*-positive women and 17% for those with *BRCA2*^4^. When breast cancer does occur among this population, those with *BRCA1* mutations are more likely to be diagnosed with triple-negative breast cancer, associated with higher risk of mortality^5^, while *BRCA2*-positive women are more likely to be diagnosed with hormone receptor-positive tumors^6^. The rate of recurrence has been approximated between 25 and 30% remains elevated among *BRCA1/2*-positive cancer survivors but remains highly dependent on individual clinical characteristics such as stage at diagnosis, treatment(s), and hormone receptor status^6^. Prophylactic treatment(s), such as hysterectomy, bilateral mastectomy, salpingectomy, and oophorectomy surgeries, remain the gold standard for preventing breast and ovarian cancers among women with these mutations^7,8^, but biannual ongoing surveillance (e.g., self-examination, magnetic resonance imaging, transvaginal ultrasound, mammogram, etc.) has also been recommended^9^.

Recently, the coronavirus disease 2019 (COVID-19) caused delays in diagnostic investigation, surgical procedures, and routine surveillance for all women, due to limited in-person services^10,11^. Many of these limitations will have long-lasting consequences such as later-stage diagnoses and poorer clinical outcomes^12^, particularly for those with *BRCA1/2* mutations, who rely on ongoing care for risk reduction and early detection. Individuals with cancer may be at greater risk of COVID-19 complications and death, worsened by older age (≥ 60 years), a history of smoking, obesity, hypertension, cardiovascular disease, and diabetes^13–15^. The COVID-19 pandemic is expected to have an increase in cancer-related mortality due to care disturbances across the cancer continuum, including but not limited to: (1) reduced access to care due to fear of infection, reallocation of resources, unemployment in the healthcare field, clinic shutdowns; (2) delayed routine care involving preventive screening, abnormal screening and symptom follow-up; (3) later-stage diagnosis indicative of reduced survival, fewer treatment options, and more invasive treatment; and (4) delayed or modified treatments like postponement of treatments and surgeries^16^. Cancer screening during the pandemic decreased 29–36% from pre-pandemic levels, and specifically, one study found an 86–94% decline in screening for breast and cervical cancers than 2017–2019 historical averages^17^. The impact of the COVID-19 pandemic on mental health outcomes among high-risk women, a population that already experience high rates of anxiety and depression^18–20^, has not yet been investigated.

Coupled with COVID-19 pandemic stressors, the impending risk of cancer, the push for prophylactic surgeries, continuous surveillance, and the associated worry of affected family members have been associated with even more increases in adverse mental health symptomology^8,21–24^ and reduced health-related quality of life^8,25,26^. Adverse mental health symptomology is often heightened considering *BRCA1/2* diagnoses and what they mean for their health in the future^24,27,28^, often compounded if testing is prompted by a cancer diagnosis^29^. Anxiety and depression are often reported among women with *BRCA1/2* mutations, most commonly among those who are undergoing genetic testing^19^, undergoing prophylactic surgeries^24^, and/or during biannual surveillance appointments^30^. Although genetic testing offers preventive opportunities and the knowledge for risk reduction and/or management, it has also been linked to increased anxiety, stress, and depressive symptomology^18,24^, trending at varying levels throughout this process.

### Objectives

The current study aims to determine the association between several COVID-19 pandemic experiences and anxiety and depression symptomology, while adjusting for covariates among *BRCA1/2*-positive US women from medically underserved backgrounds. Secondarily, we stratified these associations by income status and race/ethnicity to identify high-risk groups of mutation carriers. The importance of this paper remains unprecedented, as those with increased risk for breast and ovarian cancer due to genetic mutations may have experienced limited preventive, diagnostic, and/or treatment-related care as the COVID-19 pandemic continues into 2021 and beyond.

## Methods

### Study design and sample

Participants were recruited through national, online support groups: *BRCA1 BRCA2* Genetic Ovarian & Breast Cancer Gene (~ 11,000 members), *BRCA* Genetic Sisters Support Group (~ 6000 members), *BRCA1* & *BRCA2* Support Group (~ 3300 members), *BRCA* Strong (~ 2500 members), *BRCA* Sisterhood of Hope (~ 1400 members), Facing Our Risk for Cancer Empowered (FORCE) message boards, Understanding *BRCA* (~ 1500 members), *BRCA* Advanced & Other Hereditary Cancers Journal Club (~ 3200 members), and *BRCA* Preventive Mastectomy & Hysterectomy Support Group (~ 900 members) from December 2020 to March 2021. One study recruitment post was posted per day within each group (*BRCA* Strong only allowed one post per week), with written permission obtained from group moderators prior to posting. The post consisted of a brief announcement introducing the study, eligibility criteria, and a link to an anonymous survey. Participants were eligible if they were 18 years or older, female, lived in the US, could read/speak in English, have undergone and tested positive for either (or both) *BRCA1* and/or *BRCA2* genetic mutations within the past 5 years, and identify with at least one medically underserved population (i.e., racial, ethnic, and/or sexual minority, person with a physical disability, those with low income, first-generation immigrant, and/or those who are chronically ill). By clicking the brief study announcement, potential participants were rerouted to an anonymous screener survey to determine eligibility, and those fitting criteria were rerouted to the full online survey via REDCap (Research Electronic Data Capture) hosted at the Johns Hopkins Bloomberg School of Public Health (JHSPH)^31,32^. Survey questions prompted participants to rate anxiety, depression, COVID-19 impact, demographic characteristics, clinical cancer and genetic testing information, prophylactic surgery and ongoing surveillance history, body satisfaction, perceived worry of cancer, cancer empowerment, health-related quality of life, discrimination, and healthcare access. Participants who completed the online survey were compensated with a $20 Amazon e-gift card. This study was approved and conducted according to the ethical standards of the JHSPH Institutional Review Board (IRB) and informed consent was obtained from all participants.

### Model variables

#### Predictor variables

To measure the impact of COVID-19, the Pandemic Stress Index^33^ was utilized within the current study. The items involving COVID-19 experiences were as follows: changes in life due to COVID-19, diagnosed with COVID-19, fear of getting or spreading COVID-19, worrying about loved ones, quarantining or isolation, caring for someone at home, working from home, lost job, changes in healthcare services, stigma or discrimination, personal financial loss, frustration/boredom, not having basic supplies, more depression, more anxiety, sleep issues, increased substance use, change in sexual activity, loneliness, confusion about COVID-19, giving to the greater good by following COVID-19 mandates, and getting emotional or financial support from loved ones. The COVID-19 experiential items were entered as predictors, with one predictor in each model. Predictors were originally dichotomous with either “no” did not experience (referent) or “yes” experienced the COVID-19-related prompt during the pandemic. Items ranged from general COVID-19 occurrences (e.g., diagnosed with COVID-19, quarantining, working from home, etc.), health-related prompts (e.g., anxiety, depression, substance use, frustration/boredom, etc.), or resource reallocation (e.g., changing travel plans, financial loss, needing financial support, etc.) (Supplementary Information).

#### Outcome assessments

To measure anxiety symptomology, the Generalized Anxiety Disorder 7-item (GAD-7) scale^34^. The GAD-7 is a 7-item, 4-point Likert scale prompting, “How often have you been bothered by the following over the past 2 weeks?” ranging from 0 (not at all sure) to 3 (nearly every day). Responses were added to create a final score which ranged from zero to 21 with clinical cutoffs for mild (zero to 5), moderate (six to 10), and severe anxiety (11+)^34^. The GAD-7 has a sensitivity of 89% and specificity of 82%, utilized as a screening tool to recommend further evaluation for those scoring in the moderate to severe range^34^. In the general population, the GAD-7 reflects good reliability (α = 0.89)^35^ and excellent within the current sample (α = 0.93). For the purposes of this study, clinical cutoffs were dichotomized for mild (referent) and moderate/severe anxiety. Moderate/severe anxiety will be discussed as “high anxiety”.

Depressive symptomology was measured using the Patient Health Questionnaire (PHQ-9) Depression Assessment^36^. The PHQ-9 is a 9-item, 4-point Likert scale asking, “Over the last 2 weeks, how often have you been bothered by the following problems?” ranging from 0 (not at all) to 3 (nearly every day). Responses were combined to create a total score which ranged from zero to 27 with clinical cutoffs for minimal (zero to four), mild (five to nine), moderate (10–14), moderately severe (15–19), and severe depression (20–27)^36^. The PHQ-9 has been used in the general population, psychiatric populations, and obstetric-gynecologic populations, with an average sensitivity of 88% and specificity of 88% for major depression^36^. In the general population, the PHQ-9 has good reliability (α = 0.86–0.89) and excellent reliability (α = 0.90) within the current sample. Within the current study, clinical cutoffs were dichotomized for minimal/mild (referent) and moderate/moderately severe/severe depression. Moderate/moderately severe/severe depression will be discussed as “more depressive symptoms”.

#### Covariates and stratifications

The following variables were included as covariates across all models: age at survey completion, number of comorbid conditions, years since genetic testing, education, marital status, race/ethnicity, income status, cancer survivor status (has a history of cancer versus no cancer history), and geographic location. Age at survey completion, number of comorbid conditions (including a past cancer diagnosis) and years since genetic testing were treated as continuous. Survivor/control status was originally dichotomous and remained as such in analysis (no cancer history [referent], cancer survivor). Race and ethnicity were two separate variables. The race variable was polynomial (American Indian or Alaska Native, Asian, Native Hawaiian or other Pacific Islander, Black or African American, non-Hispanic white (NHW), biracial or multiracial) and ethnicity was dichotomous (Hispanic or Latino, not Hispanic or Latino). These variables were combined to create a race/ethnicity variable (NHW, racial/ethnic minority) due for ease of interpretation and limited subgroup size. Income status was originally polynomial, ranging from less than $20,000 USD annually to $200,000 USD annually or more. A cutoff was created based upon the US Census Bureau^37^ dichotomizing annual household income (at least $40,000 USD annually per household [referent], below $40,000 USD annually per household). Categorical covariates were originally polynomial (education [less than high school, high school graduate or GED, some college, college graduate, some graduate school, Master’s degree, professional degree, doctoral degree], marital status [married or living as married, divorced, separated, widowed, single], geographic location [urban, suburban, rural]) but were condensed: education (some college or less [referent], college graduate or above), marital status (married/living as married [referent], not married), and geographic location (urban/suburban [referent], rural). In separate models, stratifications by income status and racial/ethnic minority status were included, however, were entered as covariates when not in use as stratifications.

### Statistical methods

All analyses were performed using Stata statistical software, version 16^38^. Frequency and percentages were analyzed to identify missingness; cases that were missing were dropped for that specific model. Missingness is outlined in Table 1. Chi-square tests for categorical variables and independent samples *t* tests for continuous variables were conducted to determine potential covariates for demographic characteristics of interest. These analyses compared characteristics by income and racial/ethnic minority status) and are included in Table 1. Adjusted odds ratios (aORs) and 95% confidence intervals (CIs) were calculated with multivariable logistic regression models to measure the association between each COVID-19 experience/predictor and outcomes (anxiety and depression), while adjusting for age at survey completion, number of comorbid conditions, years since genetic testing, education, marital status, survivor/control status, geographic location, race/ethnicity, and income status. To examine the effect of experiences during COVID-19 on anxiety and depressive symptomology by income status and racial/ethnicity among *BRCA1/2*-positive women, models were stratified by these factors and an interaction term was created for COVID-19 experience combined with income status (did/did not experience during COVID-19 × income status) and race/ethnicity (did/did not experience during COVID-19 × race/ethnicity) within appropriate models. Analyses in Table 1 were two-sided and statistical significance was indicated if *p* values were below 0.05. For multivariable logistic regression models, the level of statistical significance was restricted to *p* values less than or equal to 0.02 to account for multiple tested models.

**Table 1:** Participant demographic characteristics and disadvantaged health population factors, overall and by income status and racial/ethnic minority status.

Characteristics	Stratifications					*p* value	Total female sample (N = 211) No. (%)
	Non-Hispanic White (*n* = 142) No. (%)	Racial/Ethnic Minority (*n* = 69) No. (%)	*p* value	Average/high income* (*n* = 162) No. (%)	Low income* (*n* = 49) No. (%)		
**Disadvantaged health characteristics**							
*Disability status*							
No disability	96 (67.6)	51 (73.9)	0.350	118 (72.8)	29 (59.2)	0.068	147 (69.7)
Disability	46 (32.4)	18 (26.1)		44 (27.2)	20 (40.8)		64 (30.3)
Missing	0 (0.0)	0 (0.0)		0 (0.0)	0 (0.0)		0 (0.0)
*Sexual orientation*							
Straight or not gay	106 (74.6)	56 (81.2)	0.293	129 (79.6)	33 (67.3)	0.074	162 (76.8)
LGBTQ+ or something else	36 (25.4)	13 (18.8)		33 (20.4)	16 (32.7)		49 (23.2)
Missing	0 (0.0)	0 (0.0)		0 (0.0)	0 (0.0)		0 (0.0)
*Immigration status*							
Not a first-generation immigrant	135 (95.1)	62 (89.9)	0.153	149 (92.0)	48 (98.0)	0.140	197 (93.4)
First-generation immigrant	7 (4.9)	7 (10.1)		13 (8.0)	1 (2.0)		14 (6.6)
Missing	0 (0.0)	0 (0.0)		0 (0.0)	0 (0.0)		0 (0.0)
*Multimorbidity*							
No multimorbidity	53 (37.3)	28 (40.6)	0.648	60 (37.0)	21 (42.9)	0.463	81 (38.4)
Two or more comorbid conditions	89 (62.7)	41 (59.4)		102 (63.0)	28 (57.1)		130 (61.6)
Missing	0 (0.0)	0 (0.0)		0 (0.0)	0 (0.0)		0 (0.0)
**Covariates**							
*Age*							
49 years or younger	110 (78.6)	63 (92.6)	**0.011**	134 (83.8)	39 (81.3)	0.685	173 (83.2)
50 years or over	30 (21.4)	5 (7.4)		26 (16.0)	9 (18.4)		35 (16.8)
Missing	0 (0.0)	0 (0.0)		0 (0.0)	0 (0.0)		0 (0.0)
*Education*							
College degree or above	86 (60.6)	50 (72.5)	0.090	116 (71.6)	20 (40.8)	** < 0.001**	136 (64.5)
No college degree	56 (39.4)	19 (27.5)		46 (28.4)	29 (59.2)		75 (35.5)
Missing	0 (0.0)	0 (0.0)		0 (0.0)	0 (0.0)		0 (0.0)
*Marital status*							
Married or living as married	87 (61.3)	45 (65.2)	0.578	118 (72.8)	14 (28.6)	**< 0.001**	132 (62.6)
Other	55 (38.7)	24 (34.8)		44 (27.2)	35 (71.4)		79 (37.4)
Missing	0 (0.0)	0 (0.0)		0 (0.0)	0 (0.0)		0 (0.0)
*Cancer status*							
No cancer history	92 (64.8)	46 (66.7)	0.945	102 (63.0)	36 (73.5)	0.148	138 (65.4)
Cancer survivor	47 (33.1)	23 (33.3)		58 (35.8)	12 (24.5)		70 (33.2)
Missing	3 (2.1)	0 (0.0)		2 (1.2)	1 (2.0)		3 (1.4)
*Geographic location*							
Urban or suburban	139 (83.2)	40 (93.0)	0.107	136 (84.0)	44 (89.8)	0.311	180 (85.3)
Rural	28 (16.8)	3 (7.0)		26 (16.0)	5 (10.2)		31 (14.7)
Missing	0 (0.0)	0 (0.0)		0 (0.0)	0 (0.0)		0 (0.0)
**Predictors**							
*Changes in life due to COVID-19*							
No	24 (16.9)	10 (14.5)	0.563	25 (15.4)	9 (18.4)	0.650	34 (16.1)
Yes	106 (74.6)	56 (81.2)		125 (77.2)	37 (75.5)		162 (76.8)
Missing	12 (8.5)	3 (4.3)		12 (7.4)	3 (6.1)		15 (7.1)
*Diagnosed with COVID-19*							
No	131 (92.3)	59 (85.5)	0.125	145 (89.5)	45 (91.8)	0.633	190 (90.0)
Yes	11 (7.7)	10 (14.5)		17 (10.5)	4 (8.2)		21 (10.0)
Missing	0 (0.0)	0 (0.0)		0 (0.0)	0 (0.0)		0 (0.0)
*Fear of getting COVID-19*							
No	54 (38.0)	30 (43.5)	0.448	63 (38.9)	21 (42.9)	0.619	84 (39.8)
Yes	88 (62.0)	39 (56.5)		99 (61.1)	28 (57.1)		127 (60.2)
Missing	0 (0.0)	0 (0.0)		0 (0.0)	0 (0.0)		0 (0.0)
*Fear of spreading COVID-19*							
No	74 (52.1)	47 (68.1)	**0.027**	93 (57.4)	28 (57.1)	0.974	121 (57.3)
Yes	68 (47.9)	22 (31.9)		69 (42.6)	21 (42.9)		90 (42.7)
Missing	0 (0.0)	0 (0.0)		0 (0.0)	0 (0.0)		0 (0.0)
*Worrying about loved ones*							
No	32 (22.5)	26 (37.7)	**0.021**	50 (30.9)	8 (16.3)	**0.046**	58 (27.5)
Yes	110 (77.5)	43 (62.3)		112 (69.1)	41 (83.7)		153 (72.5)
Missing	0 (0.0)	0 (0.0)		0 (0.0)	0 (0.0)		0 (0.0)
*Quarantining and isolation*							
No	54 (38.0)	31 (44.9)	0.338	70 (43.2)	15 (30.6)	0.115	85 (40.3)
Yes	88 (62.0)	38 (55.1)		92 (56.8)	34 (69.4)		126 (59.7)
Missing	0 (0.0)	0 (0.0)		0 (0.0)	0 (0.0)		0 (0.0)
*Caring for someone at home*							
No	124 (87.3)	56 (81.2)	0.235	144 (88.9)	36 (73.5)	**0.008**	180 (85.3)
Yes	18 (12.7)	13 (18.8)		18 (11.1)	13 (26.5)		31 (14.7)
Missing	0 (0.0)	0 (0.0)		0 (0.0)	0 (0.0)		0 (0.0)
*Working from home*							
No	88 (62.0)	44 (63.8)	0.800	95 (58.6)	37 (75.5)	**0.033**	132 (62.6)
Yes	54 (38.0)	25 (36.2)		67 (41.4)	12 (24.5)		79 (37.4)
Missing	0 (0.0)	0 (0.0)		0 (0.0)	0 (0.0)		0 (0.0)
*Lost job due to COVID-19*							
No	129 (90.8)	64 (92.8)	0.642	153 (94.4)	40 (81.6)	**0.005**	193 (91.5)
Yes	13 (9.2)	5 (7.2)		9 (5.6)	9 (18.4)		18 (8.5)
Missing	0 (0.0)	0 (0.0)		0 (0.0)	0 (0.0)		0 (0.0)
*Changes in healthcare services*							
No	105 (73.9)	51 (73.9)	0.996	127 (78.4)	29 (59.2)	**0.007**	156 (73.9)
Yes	37 (26.1)	18 (26.1)		35 (21.6)	20 (40.8)		55 (26.1)
Missing	0 (0.0)	0 (0.0)		0 (0.0)	0 (0.0)		0 (0.0)
*Stigma or discrimination*							
No	129 (90.8)	64 (92.8)	0.642	149 (92.0)	44 (89.8)	0.632	193 (91.5)
Yes	13 (9.2)	5 (7.2)		13 (8.0)	5 (10.2)		18 (8.5)
Missing	0 (0.0)	0 (0.0)		0 (0.0)	0 (0.0)		0 (0.0)
*Personal financial loss*							
No	97 (68.3)	53 (76.8)	0.201	126 (77.8)	24 (49.0)	** < 0.001**	150 (71.1)
Yes	45 (31.7)	16 (23.2)		36 (22.2)	25 (51.0)		61 (28.9)
Missing	0 (0.0)	0 (0.0)		0 (0.0)	0 (0.0)		0 (0.0)
*Frustration or boredom*							
No	82 (57.7)	35 (50.7)	0.336	92 (56.8)	25 (51.0)	0.476	117 (55.5)
Yes	60 (42.3)	34 (49.3)		70 (43.2)	24 (49.0)		94 (44.5)
Missing	0 (0.0)	0 (0.0)		0 (0.0)	0 (0.0)		0 (0.0)
*Not having enough basic supplies*							
No	130 (91.5)	62 (89.9)	0.687	150 (92.6)	42 (85.7)	0.141	192 (91.0)
Yes	12 (8.5)	7 (10.1)		12 (7.4)	7 (14.3)		19 (9.0)
Missing	0 (0.0)	0 (0.0)		0 (0.0)	0 (0.0)		0 (0.0)
*More anxiety*							
No	73 (51.4)	32 (46.4)	0.493	82 (50.6)	23 (46.9)	0.652	105 (49.8)
Yes	69 (48.6)	37 (53.6)		80 (49.4)	26 (53.1)		106 (50.2)
Missing	0 (0.0)	0 (0.0)		0 (0.0)	0 (0.0)		0 (0.0)
*More depression*							
No	101 (71.1)	40 (58.0)	0.057	107 (66.0)	34 (69.4)	0.664	141 (66.8)
Yes	41 (28.9)	29 (42.0)		55 (34.0)	15 (30.6)		70 (33.2)
Missing	0 (0.0)	0 (0.0)		0 (0.0)	0 (0.0)		0 (0.0)
*Sleep issues*							
No	80 (56.3)	39 (56.5)	0.980	98 (60.5)	21 (42.9)	**0.029**	119 (56.4)
Yes	62 (43.7)	30 (43.5)		64 (39.5)	28 (57.1)		92 (43.6)
Missing	0 (0.0)	0 (0.0)		0 (0.0)	0 (0.0)		0 (0.0)
*Increased substance use*							
No	126 (88.7)	59 (85.5)	0.504	142 (87.7)	43 (87.8)	0.985	185 (87.7)
Yes	16 (11.3)	10 (14.5)		20 (12.3)	6 (12.2)		26 (12.3)
Missing	0 (0.0)	0 (0.0)		0 (0.0)	0 (0.0)		0 (0.0)
*Change in sexual activity*							
No	123 (86.6)	55 (79.7)	0.195	134 (82.7)	44 (89.8)	0.232	178 (84.4)
Yes	19 (13.4)	14 (20.3)		28 (17.3)	5 (10.2)		33 (15.6)
Missing	0 (0.0)	0 (0.0)		0 (0.0)	0 (0.0)		0 (0.0)
*Loneliness*							
No	91 (64.1)	37 (53.6)	0.144	101 (62.3)	27 (55.1)	0.363	128 (60.7)
Yes	51 (35.9)	32 (46.4)		61 (37.7)	22 (44.9)		83 (39.3)
Missing	0 (0.0)	0 (0.0)		0 (0.0)	0 (0.0)		0 (0.0)
*Confusion about COVID-19*							
No	132 (93.0)	64 (92.8)	0.957	150 (92.6)	46 (93.9)	0.759	196 (92.9)
Yes	10 (7.0)	5 (7.2)		12 (7.4)	3 (6.1)		15 (7.1)
Missing	0 (0.0)	0 (0.0)		0 (0.0)	0 (0.0)		0 (0.0)
*Giving to the greater good by following mandates*							
No	88 (62.0)	56 (81.2)	**0.005**	116 (71.6)	28 (57.1)	0.057	144 (68.2)
Yes	54 (38.0)	13 (18.8)		46 (28.4)	21 (42.9)		67 (31.8)
Missing	0 (0.0)	0 (0.0)		0 (0.0)	0 (0.0)		0 (0.0)
*Getting emotional support from loved ones*							
No	105 (73.9)	52 (75.4)	0.825	124 (76.5)	33 (67.3)	0.196	157 (74.4)
Yes	37 (26.1)	17 (24.6)		38 (23.5)	16 (32.7)		54 (25.6)
Missing	0 (0.0)	0 (0.0)		0 (0.0)	0 (0.0)		0 (0.0)
*Getting financial support from loved ones*							
No	126 (88.7)	64 (92.8)	0.360	156 (96.3)	34 (69.4)	** < 0.001**	190 (90.0)
Yes	16 (11.3)	5 (7.2)		6 (3.7)	15 (30.6)		21 (10.0)
Missing	0 (0.0)	0 (0.0)		0 (0.0)	0 (0.0)		0 (0.0)

	Mean (*SD*)	Mean (*SD*)	*p* value	Mean (*SD*)	Mean (*SD*)	*p* value	Mean (*SD*)
*Age at survey completion*	40.5 (11.5)	37.5 (7.91)	0.052	39.9 (9.69)	38.2 (13.2)	0.308	39.5 (10.6)
*Number of comorbid conditions*	2.13 (1.37)	1.71 (1.12)	**0.027**	1.95 (1.29)	2.12 (1.36)	0.453	1.99 (1.31)
*Years since genetic testing*	1.96 (1.56)	1.87 (1.68)	0.694	1.99 (1.64)	1.73 (1.43)	0.300	1.93 (1.60)

LGBTQ+ = lesbian, gay, bisexual, transgender, queer, or questioning, or other.

Disadvantaged health characteristics also include income status and racial/ethnic minority status.

Bolded font indicates significant *p* value (< 0.05).

*Income status = annual household income less than $40,000 USD.

### Ethics approval

This study was performed in line with the principles of the Declaration of Helsinki. Approval was granted by the Johns Hopkins Bloomberg School of Public Health Institutional Review Board.

### Consent to participate

Informed consent was obtained from all individual participants included in the study.

## Results

### Characteristics of the study sample

Description of the study population and characteristics are shown in Table 1. A total of 211 *BRCA1/2*-positive women, both with and without a history of cancer meeting inclusion criteria were included in the current study. The sample ranged in age from 18 to 75 (*M* = 39.5, *SD* = 10.6) and most women did not have a history of cancer (*n* = 138, 65.4%). The composition of originally polynomial predictor variables was as follows: race (American Indian or Alaska Native [2.4%], Asian [6.6%], Native Hawaiian/other Pacific Islander [0.9%], Black or African American [4.3%], NHW [79.1%], biracial or multiracial [6.2%]) and ethnicity (not Hispanic/Latino [80.6%], Hispanic/Latino [18.5%]). Most of the current sample completed a college degree or above (64.5%) and was married or living as married (62.6%). Some of the women did report having at least one physical disability (40.8%) and most reported having more than one comorbid condition including cancer (61.6%). Most women reported ≥ $40,000/year for their household incomes (77%) A total of 49 participants identified as being lesbian, gay, bisexual, transgender, queer/questioning, or something else (LGBTQ+). Study characteristics among NHW and racial/ethnic minority women differed significantly. Racial/ethnic minority women were more often 49 years of age or younger (*p* = 0.011), whereas NHW women had reported significantly more comorbid conditions than racial/ethnic minority women (*p* = 0.027). Some demographic characteristics also differed significantly by income status. Women with household incomes ≥ $40,000/year more often reported a college degree or above (*p* ≤ 0.001) and being married or lived as married (*p* ≤ 0.001). COVID-19 experiences also differed by income status and racial/ethnic minority status, as depicted in Table 1.

### Anxiety and COVID-19 experiences by income status and race/ethnicity

Table 2 depicts the associations between specific COVID-19 experiences and odds of reporting high anxiety overall and stratified by income status. In adjusted models overall, women who reported experiencing depression during COVID-19 were 5.02 times more likely to report higher anxiety (95% CI [2.33, 10.8]) than women without depression. Similarly, women reporting sleep issues during this time were also significantly more likely to experience higher anxiety (aOR, 2.77, 95% CI [1.39, 5.54]) than women who did not report sleep issues. In stratified analyses by income, lower income women who reported having depression were 7.06 times more likely (95% CI [2.78, 17.9]) to also report higher anxiety compared with average/high income women (aOR, 1.99, 95% CI [0.47, 8.39]). Women of lower income were also significantly more likely to report higher anxiety (aOR, 3.65, 95% CI [1.64, 8.11]) if they reported having sleep issues during COVID-19 compared to women of average/high income (aOR, 1.12, 95% CI [0.28, 4.42]). No interactions between high anxiety and income on any COVID-19 predictor.

**Table 2:** Adjusted odds ratios (aOR) and 95% confidence intervals (CI) for the association between COVID-19 experiences and odds of high anxiety among *BRCA1/2*-positive women from disadvantaged health populations, overall and by income status and race/ethnicity.

	All women and covariates (N = 190)		Low income (N = 44)		Average/high income (N = 146)		*p* int	Racial/ethnic minority (N = 64)		NHW (N = 126)		*p* int
	aOR	95% CI	aOR	95% CI	aOR	95% CI		aOR	95% CI	aOR	95% CI	
**Changes in life due to COVID-19**												
No (33)	1.00	Reference	1.00	Reference	1.00	Reference	0.503	1.00	Reference	1.00	Reference	0.537
Yes (157)	1.18	[0.509, 2.77]	1.40	[0.531, 3.71]	0.738	[0.144, 3.78]		1.39	[0.519, 3.74]	0.758	[0.139, 4.13]	
**Diagnosed with COVID-19**												
No (169)	1.00	Reference	1.00	Reference	1.00	Reference	0.152	1.00	Reference	1.00	Reference	0.942
Yes (21)	0.793	[0.296, 2.12]	.543	[0.182, 1.62]	4.29	[0.327, 56.3]		0.768	[0.204, 2.88]	0.828	[0.181, 3.78]	
**Fear of getting COVID-19**												
No (68)	1.00	Reference	1.00	Reference	1.00	Reference	0.983	1.00	Reference	1.00	Reference	0.167
Yes (122)	1.47	[0.750, 2.88]	1.46	[0.683, 3.13]	1.48	[0.392, 5.64]		2.03	[0.894, 4.62]	0.749	[0.229, 2.44]	
**Fear of spreading COVID-19**												
No (103)	1.00	Reference	1.00	Reference	1.00	Reference	0.787	1.00	Reference	1.00	Reference	0.642
Yes (87)	1.16	[0.615, 2.20]	1.22	[0.593, 2.50]	0.993	[0.265, 3.71]		1.28	[0.603, 2.72]	0.915	[0.277, 3.02]	
**Worrying about loved ones**												
No (42)	1.00	Reference	1.00	Reference	1.00	Reference	0.268	1.00	Reference	1.00	Reference	0.835
Yes (148)	1.18	[0.541, 2.61]	.993	[0.424, 2.32]	4.13	[0.375, 45.4]		1.27	[0.448, 3.65]	1.07	[0.320, 3.62]	
**Quarantining/Isolation**												
No (67)	1.00	Reference	1.00	Reference	1.00	Reference	0.215	1.00	Reference	1.00	Reference	0.765
Yes (123)	0.919	[0.481, 1.75]	1.13	[0.550, 2.35]	0.403	[0.093, 1.75]		0.988	[0.444, 2.19]	0.795	[0.251, 2.52]	
**Caring for someone at home**												
No (162)	1.00	Reference	1.00	Reference	1.00	Reference	0.344	1.00	Reference	1.00	Reference	**0.011**
Yes (28)	1.29	[0.516, 3.25]	.910	[0.288, 2.87]	2.31	[0.500, 10.7]		3.70	[1.01, 13.5]	0.348	[0.093, 1.30]	
**Working from home**												
No (112)	1.00	Reference	1.00	Reference	1.00	Reference	0.074	1.00	Reference	1.00	Reference	0.050
Yes (78)	1.01	[0.515, 1.94]	1.39	[0.650, 2.98]	0.293	[0.064, 1.33]		1.52	[0.692, 3.34]	0.387	[0.121, 1.24]	
**Lost job due to COVID-19**												
No (172)	1.00	Reference	1.00	Reference	1.00	Reference	0.624	1.00	Reference	1.00	Reference	0.488
Yes (18)	0.991	[0.335, 2.93]	1.26	[0.286, 5.61]	0.724	[0.136, 3.85]		0.784	[0.219, 2.80]	1.99	[0.193, 20.5]	
**Change in healthcare services**												
No (135)	1.00	Reference	1.00	Reference	1.00	Reference	0.021	1.00	Reference	1.00	Reference	**0.022**
Yes (55)	0.798	[0.393, 1.61]	1.38	[0.579, 3.29]	0.209	[0.053, .823]		0.436	[0.179, 1.05]	3.12	[0.743, 13.1]	
**Stigma or discrimination**												
No (172)	1.00	Reference	1.00	Reference	1.00	Reference	0.593	1.00	Reference	1.00	Reference	0.199
Yes (18)	4.90	[1.22, 19.7]	3.70	[0.707, 19.3]	8.48	[0.685, 105.0]		**7.71**	**[1.44, 41.0]**	1.18	[0.117, 11.9]	
**Personal financial** **loss**												
No (129)	1.00	Reference	1.00	Reference	1.00	Reference	0.454	1.00	Reference	1.00	Reference	0.990
Yes (61)	0.902	[0.452, 1.79]	1.06	[0.469, 2.40]	0.593	[0.161, 2.17]		0.899	[0.397, 2.03]	0.908	[0.255, 3.23]	
**Frustration/Boredom**												
No (99)	1.00	Reference	1.00	Reference	1.00	Reference	0.515	1.00	Reference	1.00	Reference	0.904
Yes (91)	1.13	[0.591, 2.17]	1.26	[0.609, 2.61]	0.774	[0.206, 2.90]		1.10	[0.513, 2.38]	1.20	[0.377, 3.82]	
**Not having basic supplies**												
No (171)	1.00	Reference	1.00	Reference	1.00	Reference	0.206	1.00	Reference	1.00	Reference	Invariable
Yes (19)	3.68	[1.06, 12.7]	9.15	[1.07, 78.2]	1.46	[0.238, 9.01]		2.58	[0.655, 10.0]	–	–	
**More depression**												
No (123)	1.00	Reference	1.00	Reference	1.00	Reference	0.144	1.00	Reference	1.00	Reference	0.446
Yes (67)	**5.02**	**[2.33, 10.8]**	**7.06**	**[2.78, 17.9]**	1.99	[0.472, 8.39]		**4.15**	**[1.69, 10.1]**	**7.94**	**[1.88, 33.5]**	
**Sleep issues**												
No (101)	1.00	Reference	1.00	Reference	1.00	Reference	0.139	1.00	Reference	1.00	Reference	0.803
Yes (89)	**2.77**	**[1.39, 5.54]**	**3.65**	**[1.64, 8.11]**	1.12	[0.288, 4.42]		**2.63**	**[1.18, 5.88]**	3.16	[0.924, 10.8]	
**Increased substance use**												
No (164)	1.00	Reference	1.00	Reference	1.00	Reference	0.291	1.00	Reference	1.00	Reference	0.809
Yes (26)	3.01	[1.07, 8.45]	4.41	[1.16, 16.6]	1.31	[0.209, 8.24]		2.79	[0.854, 9.15]	3.78	[0.434, 33.0]	
**Change in sexual activity**												
No (158)	1.00	Reference	1.00	Reference	1.00	Reference	0.450	1.00	Reference	1.00	Reference	0.295
Yes (32)	2.56	[0.990, 6.64]	3.10	[1.03, 9.32]	1.28	[0.174, 9.49]		1.80	[0.589, 5.49]	6.57	[0.762, 56.6]	
Loneliness												
No (108)	1.00	Reference	1.00	Reference	1.00	Reference	0.870	1.00	Reference	1.00	Reference	0.602
Yes (82)	0.945	[0.497, 1.79]	.973	[0.468, 2.02]	0.861	[0.238, 3.10]		1.05	[0.489, 2.28]	0.735	[0.235, 2.30]	
**Confusion about COVID-19**												
No (175)	1.00	Reference	1.00	Reference	1.00	Reference	0.289	1.00	Reference	1.00	Reference	0.817
Yes (15)	1.89	[0.524, 6.82]	2.98	[0.581, 15.3]	0.528	[0.034, 8.13]		2.08	[0.451, 9.59]	1.50	[0.148, 15.1]	
**Giving to greater good by following mandates**												
No (124)	1.00	Reference	1.00	Reference	1.00	Reference	0.510	1.00	Reference	1.00	Reference	0.791
Yes (66)	0.798	[0.407, 1.56]	.912	[0.416, 2.00]	0.551	[0.151, 2.01]		0.760	[0.353, 1.63]	0.943	[0.229, 3.88]	
**Getting emotional support from loved ones**												
No (138)	1.00	Reference	1.00	Reference	1.00	Reference	0.199	1.00	Reference	1.00	Reference	0.560
Yes (52)	1.29	[0.631, 2.63]	1.72	[0.732, 4.05]	0.606	[0.155, 2.37]		1.46	[0.636, 3.36]	0.928	[0.251, 3.43]	
**Getting financial support from loved ones**												
No (169)	1.00	Reference	1.00	Reference	1.00	Reference	0.127	1.00	Reference	1.00	Reference	0.213
Yes (21)	0.632	[0.212, 1.88]	1.92	[0.304, 12.1]	0.315	[0.075, 1.32]		0.896	[0.268, 2.98]	0.217	[0.029, 1.62]	

Missing values: anxiety (15), change in life due to COVID-19 (15), age (3), and cancer status (3).

Bold font indicates statistically significant with corresponding *p* < 0.02.

*p* interaction terms are between income status, racial/ethnic minority status, and predictor(s).

Covariates/stratifications: age (continuous), number of comorbid conditions (continuous), years since genetic testing (continuous), education (some college or less, college graduate or above), marital status (married/living as married, other), cancer status (no cancer history, cancer history), income status (average/high income, low income), geographic location (urban/suburban, rural), and race/ethnicity (non-Hispanic white [NHW], Hispanic or racial minority).

Table 2 also shows the relationship between several COVID-19 experiences and the odds of reporting high anxiety stratified by race/ethnicity. Racial/ethnic minority women experiencing stigma or discrimination due to COVID-19 were 7.71 times more likely to report high anxiety (95% CI [1.44, 41.0]) compared to NHW women (aOR, 1.18, 95% CI [0.12, 11.9]; *p* interaction = 0.19). Racial/ethnic minority women also were more likely to report high anxiety if they experienced sleep issues during the COVID-19 pandemic (aOR, 2.63, 95% CI [1.18, 5.88]) while this association among NHW women was not significant (aOR, 3.16, 95% CI [0.92, 10.8], *p* interaction = 0.80). NHW women reported significantly with depression also had higher odds of high anxiety (aOR, 7.94, 95% CI [1.88, 33.5]) as did racial/ethnic minority women (aOR, 4.15, 95% CI [1.69, 10.1]) compared to their respective referent category who did not report depression. Racial/ethnic minority women caring for someone at home during COVID-19 were 3.70 times more likely (95% CI [1.01, 13.5]) to report high anxiety; however, this finding was not statistically significant (*p* value > 0.02). NHW women were less likely to have high anxiety with caring for someone at home (aOR, 0.34, 95% CI [0.09, 1.30], *p* interaction = 0.01). There was an additional significant interaction between changes in healthcare services and race/ethnicity (*p* interaction = 0.02); however, aORs were not statistically significant. No other interactions were observed by race/ethnicity.

### Depression and COVID-19 experiences by income status and race/ethnicity

Table 3 presents the association between the COVID-19-related experiences and the odds of reporting more depressive symptoms, overall and by income status and race/ethnicity. Women reporting stigma or discrimination (aOR, 5.14, 95% CI [1.55, 17.0]) or sleep issues (aOR, 2.52, 95% CI [1.24, 5.13]) during the COVID-19 pandemic were significantly more likely to have more symptoms of depression than women who did not experience these experiences. In models stratified by income status, women with lower incomes who reported sleep issues during the pandemic (aOR, 3.16, 95% CI [1.44, 6.94]) were significantly more likely to report increased depressive symptoms in comparison with women of average/high income (aOR, 0.96, 95% CI [0.21, 4.25], *p* interaction = 0.15). There was a significant interaction between women who reported quarantining/isolation and low income status (*p* interaction = 0.01), where women with higher incomes were significantly less likely to have depressive symptoms (aOR, 0.08, 95% CI [0.01, 0.43]) than women of lower incomes (aOR, 0.73, 95% CI [0.35, 1.52]). No other significant interactions were observed by income.

**Table 3:** Adjusted odds ratios (OR, aOR) and 95% confidence intervals (CI) for the association between COVID-19 experiences and odds of more depressive symptoms among *BRCA1/2*-positive women from disadvantaged health populations, overall and by income status and race/ethnicity.

	All women (N = 194)		Low income (N = 45)		Average/high income (N = 149)		*p* int	Racial/ethnic minority (N = 65)		NHW (N = 129)		*p* int
	aOR	95% CI	aOR	95% CI	aOR	95% CI		aOR	95% CI	aOR	95% CI	
**Changes in life due to COVID-19**												
No (34)	1.00	Reference	1.00	Reference	1.00	Reference	0.296	1.00	Reference	1.00	Reference	0.784
Yes (160)	0.877	[0.373, 2.05]	1.11	[0.423, 2.95]	0.403	[0.074, 2.17]		0.811	[0.292, 2.24]	1.03	[0.243, 4.39]	
**Diagnosed with COVID-19**												
No (173)	1.00	Reference	1.00	Reference	1.00	Reference	0.131	1.00	Reference	1.00	Reference	0.705
Yes (21)	1.31	[0.487, 3.56]	0.884	[0.289, 2.70]	8.28	[0.580, 118.3]		1.58	[0.403, 6.22]	1.07	[0.256, 4.52]	
**Fear of getting COVID-19**												
No (69)	1.00	Reference	1.00	Reference	1.00	Reference	0.844	1.00	Reference	1.00	Reference	0.045
Yes (125)	0.806	[0.412, 1.57]	0.834	[0.392, 1.77]	0.711	[0.172, 2.93]		0.468	[0.199, 1.09]	1.90	[0.651, 5.55]	
**Fear of spreading COVID-19**												
No (105)	1.00	Reference	1.00	Reference	1.00	Reference	0.893	1.00	Reference	1.00	Reference	0.549
Yes (89)	0.659	[0.342, 1.26]	0.673	[0.326, 1.38]	0.604	[0.143, 2.54]		0.569	[0.252, 1.28]	0.866	[0.286, 2.62]	
**Worrying about loved ones**												
No (43)	1.00	Reference	1.00	Reference	1.00	Reference	0.799	1.00	Reference	1.00	Reference	0.106
Yes (151)	1.00	[0.453, 2.20]	0.964	[0.417, 2.22]	1.34	[0.120, 14.9]		0.521	[0.174, 1.56]	1.91	[0.628, 5.81]	
**Quarantining/Isolation**												
No (69)	1.00	Reference	1.00	Reference	1.00	Reference	**0.019**	1.00	Reference	1.00	Reference	0.734
Yes (125)	0.491	[0.253, .954]	0.730	[0.350, 1.52]	**0.084**	**[0.016, .437]**		0.448	[0.192, 1.04]	0.570	[0.192, 1.68]	
**Caring for someone at home**												
No (164)	1.00	Reference	1.00	Reference	1.00	Reference	0.233	1.00	Reference	1.00	Reference	**0.003**
Yes (30)	1.11	[0.453, 2.72]	1.67	[0.544, 5.12]	0.497	[0.097, 2.52]		3.52	[1.11, 11.0]	0.245	[0.061, .977]	
**Working from home**												
No (115)	1.00	Reference	1.00	Reference	1.00	Reference	0.185	1.00	Reference	1.00	Reference	0.117
Yes (79)	0.924	[0.470, 1.81]	1.16	[0.546, 2.48]	0.309	[0.051, 1.86]		1.35	[0.593, 3.10]	0.467	[0.156, 1.39]	
**Lost job due to COVID-19**												
No (176)	1.00	Reference	1.00	Reference	1.00	Reference	0.155	1.00	Reference	1.00	Reference	0.178
Yes (18)	1.28	[0.423, 3.87]	2.53	[0.575, 11.1]	0.354	[0.036, 3.44]		0.723	[0.171, 3.06]	4.68	[0.456, 48.2]	
**Change in healthcare services**												
No (139)	1.00	Reference	1.00	Reference	1.00	Reference	0.251	1.00	Reference	1.00	Reference	0.070
Yes (55)	0.709	[0.337, 1.48]	0.912	[0.389, 2.13]	0.340	[0.078, 1.48]		0.389	[0.140, 1.08]	1.65	[0.504, 5.44]	
**Stigma or discrimination**												
No (176)	1.00	Reference	1.00	Reference	1.00	Reference	0.815	1.00	Reference	1.00	Reference	0.535
Yes (18)	**5.14**	**[1.55, 17.0]**	5.69	[1.29, 25.1]	4.15	[0.482, 35.5]		**6.20**	**[1.60, 24.0]**	2.66	[0.266, 26.6]	
**Personal financial loss**												
No (133)	1.00	Reference	1.00	Reference	1.00	Reference	0.122	1.00	Reference	1.00	Reference	0.654
yes (61)	1.02	[0.505, 2.06]	1.41	[0.627, 3.17]	0.391	[0.094, 1.61]		1.14	[0.483, 2.72]	0.821	[0.250, 2.69]	
**Frustration/Boredom**												
No (100)	1.00	Reference	1.00	Reference	1.00	Reference	0.100	1.00	Reference	1.00	Reference	0.922
Yes (94)	1.58	[0.813, 3.07]	2.04	[0.979, 4.27]	0.546	[0.129, 2.30]		1.54	[0.682, 3.48]	1.64	[0.558, 4.87]	
**Not** **having basic supplies**												
No (175)	1.00	Reference	1.00	Reference	1.00	Reference	0.872	1.00	Reference	1.00	Reference	0.514
Yes (19)	2.80	[0.950, 8.26]	2.61	[0.670, 10.2]	3.16	[0.504, 19.8]		2.19	[0.590, 8.14]	5.19	[0.552, 48.8]	
**More anxiety**												
No (89)	1.00	Reference	1.00	Reference	1.00	Reference	0.303	1.00	Reference	1.00	Reference	0.289
Yes (105)	1.90	[0.965, 3.74]	2.25	[1.05, 4.81]	0.980	[0.235, 4.08]		1.45	[0.635, 3.34]	3.02	[1.00, 9.10]	
**Sleep issues**												
No (89)	1.00	Reference	1.00	Reference	1.00	Reference	0.155	1.00	Reference	1.00	Reference	**0.019**
Yes (105)	**2.52**	**[1.24, 5.13]**	**3.16**	**[1.44, 6.94]**	0.961	[0.217, 4.25]		1.40	[0.598, 3.29]	**7.77**	**[2.31, 26.1]**	
**Increased substance use**												
No (103)	1.00	Reference	1.00	Reference	1.00	Reference	0.118	1.00	Reference	1.00	Reference	0.096
Yes (91)	2.19	[0.866, 5.54]	3.32	[1.12, 9.78]	0.406	[0.036, 4.56]		1.15	[0.374, 3.85]	9.43	[1.07, 82.8]	
**Change in sexual activity**												
No (168)	1.00	Reference	1.00	Reference	1.00	Reference	0.448	1.00	Reference	1.00	Reference	0.689
Yes (26)	1.76	[0.758, 4.12]	1.52	[0.604, 3.84]	3.63	[0.468, 28.1]		1.52	[0.505, 4.63]	2.18	[0.567, 8.39]	
**Loneliness**												
No (161)	1.00	Reference	1.00	Reference	1.00	Reference	0.312	1.00	Reference	1.00	Reference	0.562
Yes (33)	1.99	[1.03, 3.85]	2.36	[1.12, 4.97]	1.06	[0.265, 4.23]		1.72	[0.755, 3.92]	2.56	[0.871, 7.56]	
**Confusion about COVID-19**												
No (111)	1.00	Reference	1.00	Reference	1.00	Reference	0.832	1.00	Reference	1.00	Reference	0.453
Yes (83)	1.02	[0.311, 3.38]	0.963	[0.254, 3.64]	1.34	[0.086, 20.8]		1.46	[0.332, 6.45]	0.574	[0.082, 4.01]	
**Giving to greater good by following mandates**												
No (179)	1.00	Reference	1.00	Reference	1.00	Reference	0.378	1.00	Reference	1.00	Reference	0.851
Yes (15)	0.566	[0.278, 1.15]	0.675	[0.302, 1.50]	0.311	[0.067, 1.43]		0.591	[0.255, 1.36]	0.508	[0.133, 1.93]	
**Getting emotional support from** **loved ones**												
No (128)	1.00	Reference	1.00	Reference	1.00	Reference	0.895	1.00	Reference	1.00	Reference	0.836
Yes (66)	0.783	[0.380, 1.61]	0.803	[0.355, 1.81]	0.718	[0.162, 3.16]		0.741	[0.303, 1.80]	0.868	[0.258, 2.91]	
**Getting financial support from loved ones**												
No (140)	1.00	Reference	1.00	Reference	1.00	Reference	0.100	1.00	Reference	1.00	Reference	0.268
Yes (54)	1.21	[0.387, 3.78]	4.05	[0.625, 26.3]	0.536	[0.117, 2.45]		1.67	[0.480, 5.82]	0.467	[0.060, 3.59]	

Missing values: change in life due to COVID-19 (15), age (3), and cancer status (3).

Bold font indicates statistically significant with corresponding *p* < 0.02.

*p* interaction terms are between income status and predictor(s).

Covariates/stratifications: age (continuous), number of comorbid conditions (continuous), years since genetic testing (continuous), education (some college or less, college graduate or above), marital status (married/living as married, other), cancer status (no cancer history, cancer history), income status (average/high income, low income), geographic location (urban/suburban, rural), and race/ethnicity (non-Hispanic white [NHW], Hispanic or racial minority).

In models stratified by race/ethnicity (Table 3), minority women who experienced stigma or discrimination related to COVID-19 had 6.20 times the odds of reporting more depressive symptoms (95% CI [1.60, 24.0]) while NHW women had 2.66 times the odds (95% CI [0.26, 26.6]) of this outcome, although this association was not significantly different. The association between sleep issues due to the pandemic and depression symptoms was significantly modified by race/ethnicity (*p* interaction = 0.01). NHW women reporting sleep issues during the pandemic were 7.77 more likely (95% CI [2.31, 26.1]) to experience more depressive symptoms while minority women were only 1.40 times more likely (95% CI [0.59, 3.29]), although this finding among minorities was not statistically significant. There were also statistically significant racial/ethnic differences among women caring for someone at home during the COVID-19 pandemic and odds of depression. NHW women were less likely (aOR, 0.24, 95% CI [0.06, 0.97]) to report more depressive symptoms, while racial/ethnic minority women were 3.52 times more likely (95% CI [1.11, 11.0]) to experience depression symptomology, although neither relationship was significant (*p* interaction = 0.003). No other significant interactions were observed by race/ethnicity.

## Discussion

Among *BRCA1/2*-positive women residing in the US, the current study analyzed relationships between experiencing COVID-19-related instances and odds of reporting anxiety and depression overall and stratified by sociodemographic factors. Historically, women with *BRCA1/2* mutations have been difficult to recruit in large numbers, partly due to the rarity of the prevalence of these mutations. In past literature, women with these mutations have been primarily recruited from hospital genetics programs or gynecologists offices in small numbers^39^. Due to the nature of the COVID-19 pandemic and with hospital outpatient non-essential care halting, the current study piloted recruitment through online Facebook *BRCA1/2*-oriented support groups, which were very successful in reaching our recruitment ceiling of 225 consented participants in 3.5 months.

Demographically, most of the sample was younger than age 50, consistent with past literature suggesting that women are being genetically tested at younger ages^40,41^. Most women were NHW and educated, but there was some diversity where as much as 40.8% reported a physical disability and 61.6% a chronic condition. The current study is novel in its relation to COVID-19, however research remains limited regarding the pandemic and its impact on at-risk cancer populations such as those with *BRCA1/2* mutations. Commonalities existed with several COVID-19-related experiences predicting increases in anxiety and depression symptomologies among women with *BRCA1/2* mutations. It appears reporting stigma or discrimination or sleep issues during the pandemic resulted in significantly increased chances of having more anxiety and depression symptoms than women who did not report these instances. Although it is well-known that *BRCA1/2*-positive women report on average, higher levels of anxiety and depression than the general population, these increases have not been directly connected to the COVID-19 pandemic, but within past literature have focused on the stress of ongoing surveillance and prophylactic risk-reducing surgeries^18,42,43^ and cancer patients generally^44^. Among women of the general population, previous literature has found that both anxiety and depression symptomologies were heightened during the pandemic, highlighting the impact on the impact of mental health in various communities^45^. Specifically, risk factors for worsened mental health distress included female gender, being below the age of 40, having additional chronic or psychiatric illnesses, unemployment, student status, and/or frequent exposure to COVID-19-related social media or news coverage^46^. Additionally, one study posited that those in fair/poor health, of below-average income, and those who knew someone infected with COVID-19 experienced higher levels of mental health distress. However, NHW individuals, those of above-average income, and those who spent less than 8 h on electronic screeners per day were likely to experience lower levels of distress^47^. While some of these risk factors may overlap with women of the current sample, future research should focus on discerning these factors from the US general female population.

Not surprisingly, there were differences in income status, where women with average/high income were less likely to report depressive symptoms if they quarantined due to COVID-19. As we know, individuals who have both the resources and time to seek mental healthcare are more likely to utilize such care^48,49^, but does not account for COVID-19-related barriers. Interestingly, for those caring for someone at home during the pandemic, there were differences by race/ethnicity, where NHW were less likely to experience depression, but minority women were almost three times more likely. Past literature has found that among caregivers, depression and anxiety were higher in Black or African Americans than NHW women^50^, but other literature has reported that mental health symptoms increased with level of care^51^. It is also possible that COVID-19 experiences vary based upon geographic location within the US (urban, suburban, rural), and although not the case within the current study perhaps due to invariability, has been highlighted in past literature among the general population^45,52^ and reproductive cancer patients^53,54^, noting that those living in rural areas were more likely to experience greater anxiety than those living in urban areas. Due to the recency of the COVID-19 pandemic in conjunction with its effect on both cancer patients, survivors, and those at increased risk for cancer like the women in this study, this topic remains relatively new and suggests the importance of researching this further.

To our knowledge, no studies have been conducted focusing on *BRCA1/2*-positive women’s mental health and their relation to the COVID-19 pandemic. In current literature, healthcare utilization in relation to genetic testing^55^ and cancer-related diagnostic delays^56^ has been introduced in recent years, but not many have highlighted how the pandemic has impacted cancer patients or survivors’ mental health. In one such study, Wang and colleagues^20^ published that among 6213 cancer patients, 23.4% experienced depression and 17.7% had anxiety. In relation to COVID-19, individuals showing a history of mental health adversities, alcohol consumption, and continuous cancer worry were predominant factors for mental health symptomology among this population^20^. The recent pandemic’s impact on the mental health of the general population has been published more often, noting that both the direct and indirect psychological impact of COVID-19 on the general public and vulnerable groups (e.g., elderly, people with pre-existing mental health issues, etc.)^57^ should be studied in more detail. Similarly, symptoms of mental health during COVID-19 have been exacerbated by lower quality of life and focusing on the negative aspects of the pandemic^58^. The recency of the COVID-19 pandemic in the US has focused research on the general population and its mental health, while very little, to our knowledge, has been implemented among cancer patients or survivors, and none regarding *BRCA1/2*-positive women.

It is apparent that the COVID-19 pandemic had variable effects on certain groups such as *BRCA1/2*-positive racial/ethnic minority women and those with low income. While research is continuing to emerge in response to the COVID-19 pandemic in relation to cancer and cancer risk, future studies should focus on stratifying by groups who are at higher risk for cancer and those who have survived it. Larger, more inclusive nationwide studies may provide the framework necessary to distinctly analyze subgroups such as these so resources following this pandemic may be of benefit to all in the US. Longitudinal studies could be implemented to discover the impact of COVID-19 on the cancer care continuum, from screening to survivorship. Resources should be made available to individuals experiencing compounded disparities, like those mentioned in the current study, to help alleviate the adverse mental health symptoms that may arise due to COVID-19, surveillance, and surgery. The National Cancer Institute (NCI)^59^ and American Cancer Society (ACS)^16,60^, and even several large hospital systems such as Johns Hopkins Medicine in collaboration with the National Comprehensive Cancer Network (NCCN)^61^ have published websites to assist cancer patients and survivors navigate the COVID-19 pandemic. Clinically, mental health screening at routine healthcare appointments may be beneficial to this population in combination with available mental health resources and recommendations. However, because this is a new realm of research, additional research is needed to accurately describe the relationship between COVID-19, anxiety, and depression among at-risk cancer groups such as women with *BRCA1/2* mutations.

### Study strengths

The current study has several strengths. Our study attempted to recruit from a combination of hard-to-reach populations and those with rare cancer hereditary genetic mutations not easily recruited in-person. The online nature of this study acted as a pilot to test if these populations could be recruited successfully and from areas across the US. We were able to recruit a female sample from diverse backgrounds, allowing for limited generalizability to subpopulations such as racial/ethnic minorities, those with low income, and those with cancer. Future studies can use these approaches to recruit other hard-to-reach populations for rare or stigmatized health conditions.

### Limitations

The current study’s findings should be interpreted with consideration of its limitations. Overall, while the current study provided a moderately large sample, the data is cross-sectional and self-reported, which may introduce misclassification or recall bias. While we did collect information on the presence of physical disability, we did not gauge severity, subtype, or impact on activities of daily living (ADLs) based upon reported physical disability. We also did not collect information on COVID-19 severity or recency to include in these analyses. This study recruited *BRCA1/2*-positive women during various stages of the COVID-19 pandemic, where hospitalization, deaths, and vaccination rates varied both nationally and at community levels. We were unable to account for these variables in the current analysis, but future studies should capture these data to account for these distinctions. Stratified results should be interpreted with caution due to limited sample sizes among the subgroups of interest. Our findings should be replicated in a larger study with a similar study population to confirm similarities. It is also possible that by using predictors that were originally dichotomous may limit the implication of detailed information, as future studies may ask about the severity of COVID-19 experiences in addition to incidence. These participants were recruited from online support groups, which may introduce bias by being more open and willing to share experiences than others not in support groups^62^. Therefore, generalization of these findings is limited to the populations analyzed in the current sample.

## Conclusion

The current study provides a unique view in beginning to understand the impact of the COVID-19 pandemic on anxiety and depression among women with *BRCA1/2* mutations. This perspective allowed the identification of several COVID-19-related experiences in relation to mental health outcomes, stratified by income status and race/ethnicity, showing that there are distinct disparities among both groups. Future research can target the development of anxiety and depressive symptom relief during and after the COVID-19 pandemic utilizing prospective longitudinal study designs, while interventions can focus on recurrent training for medical professionals working with this population. Clinically, medical professionals should offer referrals to mental health counselling for all patients, not only those who are visibly struggling during this pandemic. With genetic testing becoming more widely available, especially with the utilization of telemedicine, it is possible that women may require ongoing mental healthcare that are not currently widely available for those of low income and racial/ethnic minority groups to reduce the inequities among those with *BRCA1/2* mutations.

## Supplementary Information

Supplementary Information.

## Data Availability

The datasets generated during and/or analyzed the current study are available at the Principal Investigator (PI)’s discretion upon reasonable request.

## Code availability

Syntax coding is available upon reasonable request from the corresponding author.
