# Supplementary material for: COVID-19 experiences predicting high anxiety and depression among a sample of BRCA1/BRCA2-positive women in the US
Source: Sci Rep. 2021 Dec 30;11:24501. doi: 10.1038/s41598-021-04353-x (PMC8718530; doi:10.1038/s41598-021-04353-x)
Supplement: Supplementary file 1 — Supplementary Information. [file 41598_2021_4353_MOESM1_ESM.docx]

| **Supplement A.** Instrumentation measures and model variables | | | | |
| --- | --- | --- | --- | --- |
| **MEASURE** | **ITEM/VARIABLE** | **ORIGINAL CODING** | **ANALYTIC CODING** | **TYPE OF VARIABLE** |
| Sociodemographic information | Age | Continuous | Continuous | Covariate |
|  | Number of comorbid conditions | Continuous | Continuous | Covariate |
|  | Years since genetic testing | Continuous | Continuous | Covariate |
|  | Education | 0 = Less than high school  1 = High school or GED  2 = Some college  3 = College graduate  4 = Some graduate school  5 = Master’s degree  6 = Professional degree (JD, MD)  7 = Doctoral degree | 0 = Some college or less  1 = College graduate or above | Covariate |
|  | Marital status | 0 = Married or living as married  1 = Divorced  2 = Separated  3 = Widowed  4 = Single, never married | 0 = Married or living as married  1 = Other | Covariate |
|  | Race | 0 = American Indian/Alaska Native  1 = Asian  2 = Native Hawaiian/other Pacific Islander  3 = Black or African American  4 = non-Hispanic white  5 = Biracial or multiracial | 0 = non-Hispanic white  1 = racial/ethnic minority | Covariate & Stratification |
|  | Ethnicity | 0 = Not Hispanic/Latino  1 = Hispanic/Latino |  |  |
|  | Income | 1 = Less than $20,000  2 = $20K - $39,999  3 = $40K - $74,999  4 = $75K - $99,999  5 = $100K - $149,999  6 = $150K - $199,999  7 = $200,000 + | 0 = at least $40,000 USD annually/household  1 = below $40,000 USD annually/household | Covariate & Stratification |
|  | Survivor/control status | 0 = No cancer history  1 = Cancer survivor | 0 = No cancer history  1 = Cancer survivor | Covariate |
|  | Geographic location | 0 = Urban  1 = Suburban  2 = Rural | 0 = Urban or suburban  1 = Rural | Covariate |
| Generalized Anxiety Disorder 7-item (GAD)^34^ | 1. Feeling nervous, anxious or on edge | 0 = Not at all  1 = Several days  2 = Over half the days  3 = Nearly every day  *Total score was summed and dichotomized based on clinical cutoffs reported in instrument manual | 0 = Mild  1 = Moderate or severe | Outcome |
|  | 2. Not being able to stop or control worrying |  |  |  |
|  | 3. Worrying too much about different things |  |  |  |
|  | 4. Trouble relaxing |  |  |  |
|  | 5. Being so restless that it is hard to sit still |  |  |  |
|  | 6. Becoming easily annoyed or irritable |  |  |  |
|  | 7. Feeling afraid as if something awful might happen |  |  |  |
| Patient Health Questionnaire 9-item (PHQ-9) Depression Assessment^36^ | 1. Little interest or pleasure in doing things | 0 = Not at all  1 = Several days  2 = Over half the days  3 = Nearly every day  *Total score was summed and dichotomized based on clinical cutoffs reported in instrument manual | 0 = Minimal or mild  1 = Moderate, moderately severe, or severe | Outcome |
|  | 2. Feeling down, depressed, or hopeless |  |  |  |
|  | 3. Trouble falling or staying asleep, or sleeping too much |  |  |  |
|  | 4. Feeling tired or having little energy |  |  |  |
|  | 5. Poor appetite or overeating |  |  |  |
|  | 6. Feeling bad about yourself – or that you are a failure or have let yourself or your family down |  |  |  |
|  | 7. Trouble concentrating on things, such as reading the newspaper or watching television |  |  |  |
|  | 8. Moving or speaking so slowly that other people could have noticed. Or the opposite - being so fidgety or restless that you have been moving around a lot more than usual |  |  |  |
|  | 9. Thoughts that you would be better off dead, or of hurting yourself |  |  |  |
| Pandemic Stress Index (PSI)^33^ | 1. Changes in life due to COVID-19  2. Diagnosed with COVID-19  3. Fear of getting COVID-19  4. Fear of spreading COVID-19  5. Worrying about loved ones  6. Quarantining/isolation  7. Caring for someone at home  8. Working from home  9. Lost job due to COVID-19  10. Change in healthcare services  11. Stigma or discrimination from others  12. Personal financial loss  13. Frustration/boredom  14. Not having basic supplies  15. More anxiety  16. More depression  17. Sleep issues  18. Increased substance use  19. Change in sexual activity  20. Loneliness  21. Confusion about what COVID-19 is  22. Giving to greater good by following mandates  23. Getting emotional support from loved ones  24. Getting financial support from loved ones | 0 = Did not experience  1 = Experienced | 0 = Did not experience  1 = Experienced | Predictors  (One per model) |
